# Giant basal cell carcinoma associated with vitiligo

**DOI:** 10.1002/ccr3.2359

**Published:** 2019-08-14

**Authors:** Luiza Fiszon‐Cerqueira, Marcia Ramos‐e‐Silva, Flávio Bacelar Guerreiro, Marcela Cistaro‐Serrano, Ana Helena Correia Carneiro, Maria Kátia Gomes

**Affiliations:** ^1^ Sector of Dermatology and Post Graduation Course in Dermatology – University Hospital and School of Medicine Federal University of Rio de Janeiro Rio de Janeiro Brazil; ^2^ Department of Pathology – University Hospital and School of Medicine Federal University of Rio de Janeiro Rio de Janeiro Brazil; ^3^ Department of Family Medicine – University Hospital and School of Medicine Federal University of Rio de Janeiro Rio de Janeiro Brazil

**Keywords:** basal cell carcinoma, giant basal cell carcinoma, squamous cell carcinoma, vitiligo

## Abstract

We report a woman with two giant Basal cell carcinomas (BCCs) on the back, the largest with 12 cm in diameter, inside a vitiligo plaque. They were metatypical subtype BCC associated with a nodular subtype area. Thinking of BCC in vitiligo lesion is extremely important. Giant BCC and vitiligo are rare association.

## 1 INTRODUCTION

A 65‐year‐old woman presented two ulcerations on the back, the largest measuring 12 cm in diameter, located on a vitiligo plaque. Histopathology confirmed a metatypical subtype basal cell carcinoma associated with an area of nodular subtype. Only four cases of BCC with vitiligo were found, none a giant BCC.

Basal cell carcinoma is the most prevalent skin cancer and the most widespread malignancy in humans.^1^, ^2^ BCC has a multifactorial etiology, with advanced age and greater exposure to UV radiation playing relevant roles. It grows slowly, about one centimeter per year with little tendency to local invasion and low risk of metastases.^2^ Giant BCC represents less than 1% of all BCCs and can be more invasive locally with higher risk of metastases.^1^ The nodular subtype is considered the most common among the giant BCCs, followed by the infiltrative subtype.^2^

Vitiligo is a common skin disease among the world population, characterized by achromic skin macules. The lower incidence of nonmelanoma skin cancer in patients with vitiligo has been the subject of discussions, and there is still no consensus regarding such association.

We report a case of a 65‐year‐old female patient with giant BCC with evolution of approximately 15 years and widespread vitiligo. There were only four cases found in literature reporting patients with vitiligo and basal cell carcinoma, with this being the first case of giant BCC associated with the disease.

## 2 CASE REPORT

A 65‐year‐old woman, housewife and born in Rio de Janeiro, refers a small erythematous macula on her back for the past 15 years. It evolved into a painful ulcerated lesion after 7 years that has been growing since then, recently with increasing speed. As auto‐medication, she used silver sulfadiazine intermittently. For fear of the diagnosis, patient never sought medical assistance. She has generalized nonsegmental vitiligo for 20 years and has never been submitted to phototherapy and had very mild sun exposure through her life.

**Figure 1:**
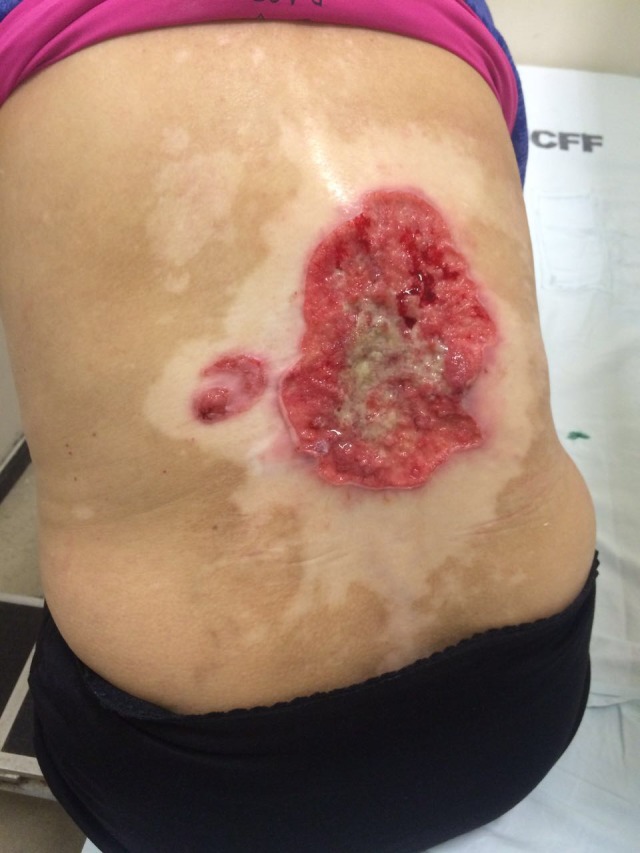
Ulcerated lesions on the back on a large achromic patch of vitiligo

**Figure 2:**
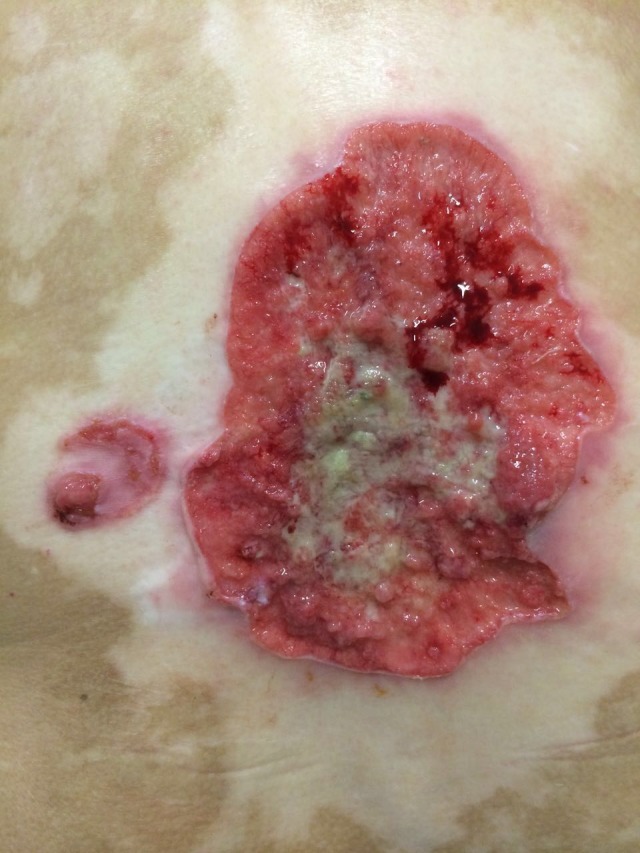
Close‐up of the lesions on the back

At examination, we observed an ulcerated lesion with a vegetating basis. There was bleeding with yellowish exudate in the center and raised borders of about 12 cm in its largest diameter. A satellite erythematous lesion of about 3 cm with minor ulceration could also be observed to the left of the main lesion. Both lesions were located on a large achromic lesion compatible with vitiligo on the dorsum (Figures 1 and 2). Examination did not reveal any palpable lymphadenomegaly.

An incisional biopsy was carried out on the border of the largest lesion that showed basal cell carcinoma of the infiltrative type.

A CT scan evidenced a lesion affecting the skin and underlying tissue, initiating at the dorsal thoracolumbar transition at T11 level, without cleavage plane with the muscular structure at T12, L1 and L2 levels, without bone involvement or lymphadenomegaly. Laboratory analyses including blood count, coagulation time, and liver function were normal.

**Figure 3:**
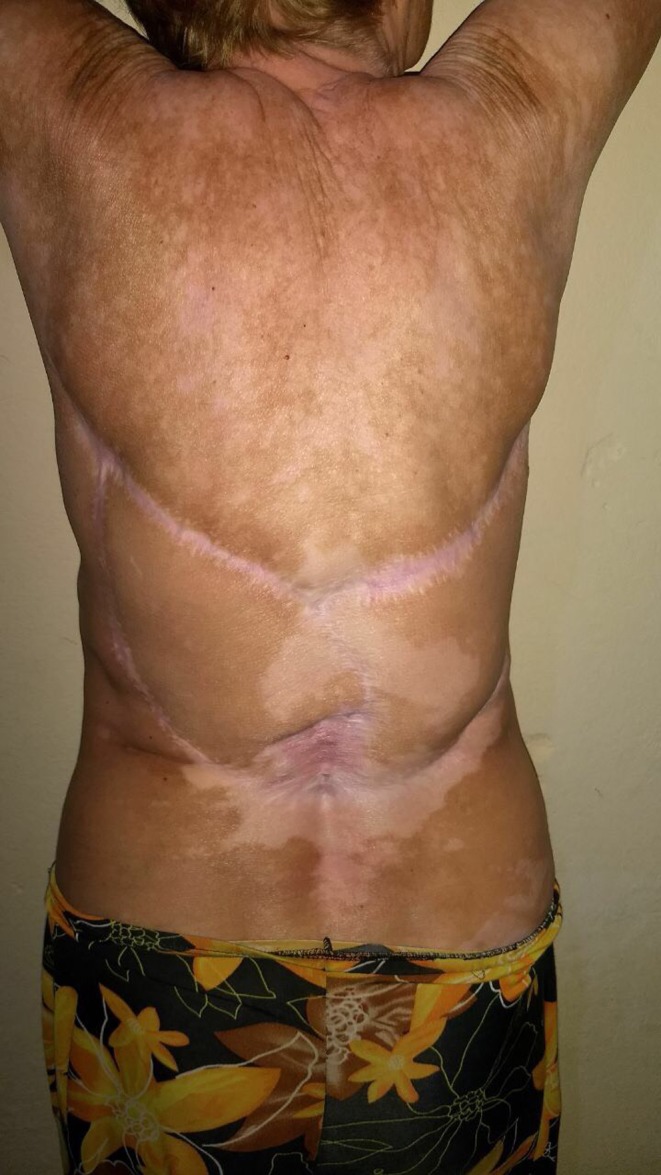
Three months after the surgery

The plastic surgery jointly with Orthopedics excised the tumor with reconstruction using the dorsal muscle and partial skin graft using the gluteus as donor area. The muscle biopsy freezing procedure during surgery revealed free surgical margins. Although it was needed for a very large excision, the surgical site had a good aspect seven months after the removal of the tumor (Figure 3).

**Figure 4:**
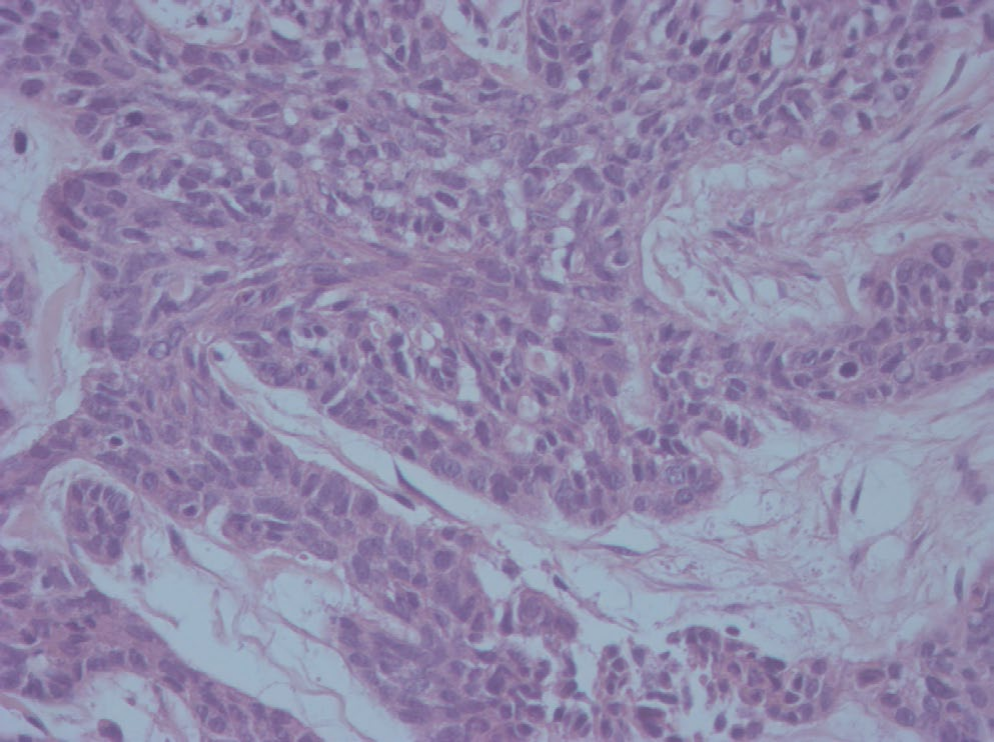
Histopathology of metatypical basal cell carcinoma showing cells with larger and more atypical nuclei, without evident peripheral palisade (HE, 400×)

**Figure 5:**
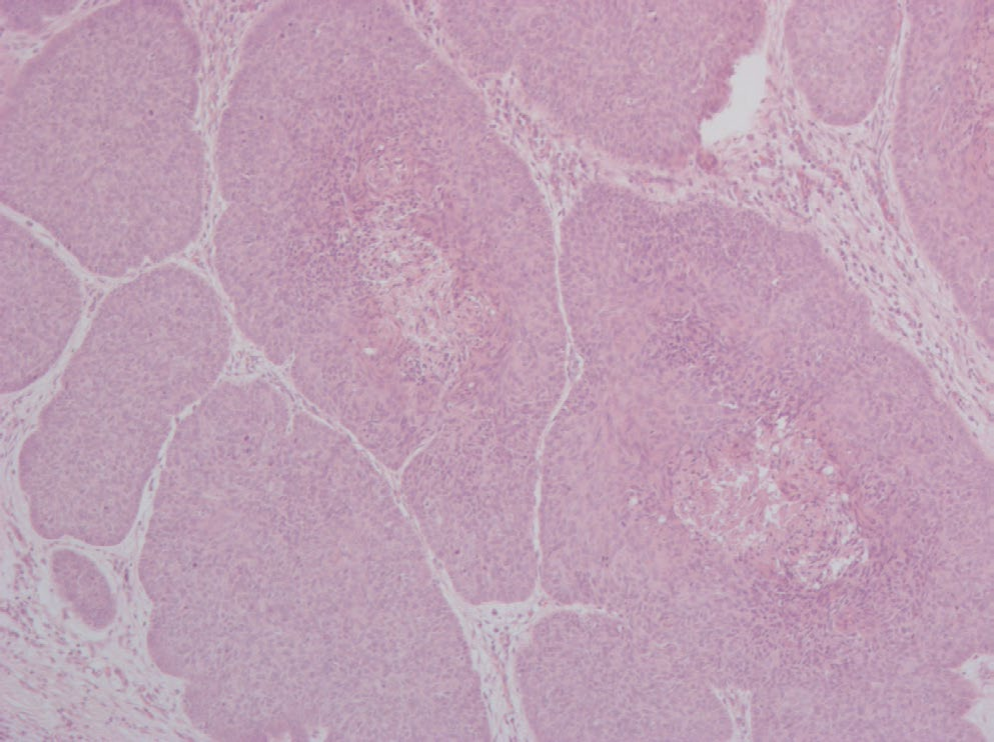
Focal area of nodular basal cell carcinoma (HE, 100×)

The excised surgical fragment measured 16.0 × 12.5 × 2.0 cm with a central ulcerated area, which measured 12.0 × 11.0 cm, and the adjacent one, measured 3.0 × 2.5 cm. The nearest limit was 1.4 cm distant from the lesion. Histopathologic examination revealed metatypic infiltrative type basal cell carcinoma (Figure 4), forming an ulcerated cutaneous tumoral lesion with an extension of 12 cm in its largest diameter. Association with the basal cell carcinoma of the nodular type was observed (Figure 5) and corresponds to around 5% of the tumoral volume present in the examined sample. The neoplasia infiltrated the subcutaneous adipose tissue.

## 3 DISCUSSION

Basal cell carcinoma is the most common cancer worldwide, usually with slow evolution and little local invasion.^2^ According to the classification of the “American Joint Committee on Cancer” (AJCC), BCC is called giant BCC when exceeding 5 cm in its largest diameter, despite some authors considering 10 cm.^1^, ^2^, ^3^, ^4^, ^5^ Giant BCCs represent from 0.4% to 1% of all BCCs^2^, ^3^ occurring with greater frequency in areas not exposed to sunlight, as dorsum, shoulders, legs, and thighs,^3^, ^4^, ^5^ with the main risk factors for their onset being negligence, whether for denying the disease, fear of the diagnosis, poor education, and Alzheimer's disease, among others.^1^, ^2^, ^3^

Despite BCC being a low‐risk tumor regarding tendency to cause metastases, ranging between 0.03%‐0.55%,^5^ the giant form usually has a more aggressive behavior with greater frequency for metastases. Tumors greater than 3 cm present a 2% chance of metastasis, and those exceeding 5 cm raise the chances to 25%; over 10 cm to 45%‐50% and those exceeding 25 cm have an almost 100% chance to present metastases.^1^, ^2^, ^3^

Histopathologically, the most aggressive BCCs (morpheiform, micronodular, and metatypical) have a greater chance to progress to giant BCCs.^2^, ^3^ However, a meta‐analysis study demonstrated that the nodular subtype is the most prevailing (53%), followed by the infiltrative (20%) and morpheiform (9%) subtypes.^6^ Perivascular and perineural invasion is relevant for tumor dissemination.^3^

In our patient, the tumor had characteristics in part of nodular BCC and in part of metatypical BCC. Despite the large size of the lesion and being of the aggressive subtype, the patient did not present signs of metastatic disease.

Metatypical BCC is considered rare, with an approximate incidence of 1.2%‐2.7% among all types of skin cancers.^7^, ^8^, ^9^ Its occurrence is more frequent in white patients and found more frequently in the head and neck.^8^, ^9^, ^10^ Both the clinic and morphology of the metatypical BCC are similar to the other BCC types, but it is more aggressive with higher tendency to metastases.^10^

Histologically, metatypical BCC is defined as a tumor that has BCC and squamous cell carcinoma (SCC) areas with a transition area between both.^9^, ^10^, ^11^ Presently, the most accepted definition is that this subtype tumor is a carcinoma comprising two typical elements: (a) Infiltrative growth of basaloid cells with scarce cytoplasm and large, uniform and pale nuclei; (b) Bundles of squamous cells with abundant eosinophilic cytoplasm located near the center or disseminated throughout the entire lesion.^7^, ^9^, ^11^

Histopathologic diagnosis of this BCC subtype is usually only established after removal of the entire lesion. A study by Leibovitch et al demonstrated that only 13.7% of the analyzed patients had a conclusive metatypical BCC diagnosis at the first biopsy, while the majority only obtained the correct diagnosis after total lesion removal, as in the case of our patient.^12^

Vitiligo is an acquired pigmentation disorder that affects 0.5%‐1% of the population.^5^, ^6^, ^7^, ^8^, ^9^, ^10^, ^11^, ^12^, ^13^ It is characterized by idiopathic destruction of melanocytes, possibly immunomediated, resulting in achromic macules.^14^ Melanin is a unique system for absorption of light and filtering of UV rays, distributed throughout the melanosomes, acting as a supranuclear tampon in the basal and suprabasal cells of the human epidermis. Its main function is considered as protecting those cells against the harmful effects of UV radiation.^13^ It would be expected that patients with vitiligo were subject to a higher skin cancer incidence and greater actinic damage because of the lack of protective effect of melanin in the skin. However, several studies demonstrated a lower nonmelanoma and melanoma skin cancer incidence in patients with vitiligo.^13^, ^14^, ^15^, ^16^

Onset of skin cancer in vitiligo patients is considered rare. Four case reports of basal cell carcinoma in vitiligo patches were found in literature, with one of them subsequent to PUVA therapy.^17^, ^18^, ^19^, ^20^ There are also a few case reports of squamous cell carcinoma in the skin of patients with vitiligo.^21^

Some studies were already carried out to evaluate the incidence of nonmelanoma skin cancer in patients with vitiligo. A study with 136 patients with vitiligo with photo exposure did not find photodamage or nonmelanoma skin cancer in any of those patients.^13^ A retrospective cohort study comprising 1307 patients also found a lower probability for development of nonmelanoma skin cancer in patients with vitiligo.^14^ Another cohort study with 10 040 patients with vitiligo presented results confirming the anterior retrospective study, showing a lower probability for development of nonmelanoma skin cancer in patients with vitiligo.^15^ Those studies, however, contradict a cohort study with 477 patients with vitiligo that found a higher, despite statistically nonsignificant incidence of nonmelanoma skin cancer in patients with vitiligo.^22^

The lower incidence of melanoma and nonmelanoma skin cancer in patients with vitiligo may be explained by an over expression of the p53 protein in normal skin and in skin affected by vitiligo.^15^, ^16^ The p53 protein regulates the expression of genes that control the progression of the cell cycle, induction of cell apoptosis and repair of DNA and functions involved in cell response to stress. The p53 protein modulates the DNA repair process through progression of the cell cycle, thus giving time for repair of the DNA damage.^16^

## 4 CONCLUSION

Basal cell carcinoma is the most common skin cancer in the world and has a tendency to be noninvasive. Giant BCC is rare and has a higher chance of metastasis. The patient of the presented case had a giant BCC with 15 years of evolution due to negligence as described in the literature.

Although several studies show a lower incidence of BCC in patients with vitiligo, one should consider the possibility of the onset of the disease in these patients and consider that it can progress to a giant BCC.

## CONFLICT OF INTEREST

None declared.

## AUTHOR CONTRIBUTIONS

Luiza Fiszon Cerqueira: collected the data, analyzed and interpreted the data, drafted the article, and finally approved the version to be published. Marcia Ramos‐e‐Silva: analyzed and interpreted the data, critically revised the article, and finally approved the version to be published. Flávio Bacelar Guerreiro: collected the data, analyzed and interpreted the data, critically revised the article, and finally approved the version to be published. Marcela Cistaro: collected the data, analyzed and interpreted the data, critically revised the article, and finally approved the version to be published. Ana Helena Correia Carneiro: collected the data, analyzed and interpreted the data, critically revised the article, and finally approved the version to be published. Maria Kátia Gomes: concepted the work, collected the data, analyzed and interpreted the data, drafted the article, critically revised the article, and finally approved the version to be published.
